# Our Enterprise

**Published:** 1840-01

**Authors:** 


### WESTERN JOURNAL.

NO. I.

LOUISVILLE, JANUARY, 1840.

### OUR ENTERPRISE.

In presenting the profession, in the Valley of the Mississippi, with the first number of a new Medical Journal, we are influenced by three facts — first, that there is not, at this time, a single periodical of the kind publishing west of the mountains,—second, that for most of the time since the year 1827, two have been supported in this part of the United States; and third, that such a work is needed by our physicians, for the double purpose of interchanging among themselves the results of their experience, and of acquiring from abroad, that information on the progress of discovery and improvement, which can only be obtained through the periodical press. That similar enterprises, with which we ourselves as well as others, have been connected, have gone forward with an interrupted movement, and even, at length, been suspended, does not in any degree discourage us; as such results are inevitable in a new country. Our motto is, NIL DESPERANDUM. We do not, however, intend to fold our arms in hope, but to rest our hopes upon them, and of course to secure success by those labours which only can command it.

Utility will be the object of our exertions, and to this end we respectfully invite the co-operation of our Western and Southern brethren. Isolated facts, reports of particular cases, histories of epidemics, bills of mortality, meteorological tables, and notices of dicovery in botany, zoology and natural history generally — every thing, in short, which can throw light upon the diseases, climatorial temperament and geological constitution of the vast intramontane platform lying between the lakes and the Gulf of Mexico, will be thankfully received and promptly inserted, if deemed of importance to the medical public. Wedded to no particular systems of pathology or therapeutics, we shall freely admit original facts and speculations of all kinds, provided they bear an aspect of truth and utility. In thus throwing open our pages we shall not hold ourselves responsible for the verity or soundness of any thing not emanating from our own pens; though we shall at all times endeavour to exclude "false facts" and frivolous speculations. Under this rule, we have inserted, in the present number, a review, by one of our most able and authoritative collaborators, of the published lectures of a distinguished eastern physician, without inquiring whether or not the opinions of our learned friend are such as we could endorse.

Our connexion, and that of some of our associates, with the LOUISVILLE MARINE HOSPITAL, in which there are generally about fifty patients, will enable us to enrich our pages with many important reports on clinical medicine and pathological anatomy, of which a specimen will be given in the next number.

We have chosen the *monthly* form, as most correspondent to the taste of the age; which under the present improved modes of distribution throughout our own country, and of intercourse with Europe, can only be satisfied with early intelligence. This, by the visit of one of our collaborators, to London and Paris, we shall secure within a few months.

It only remains for us to add, that the present number has been brought out, under circumstances which precluded us from bestowing upon it the proper degree of attention; and that, although it contains some respectable papers from our correspondents, it is not equal in its general character to our wishes, nor, as we trust, a fair specimen, of the future numbers of the WESTERN JOURNAL OF MEDICINE AND SURGERY.

